# Effect of a multicomponent school-based intervention with parental involvement on socioeconomic inequalities in smoking initiation: equity impact analysis of the TOPAS study

**DOI:** 10.1136/jech-2024-222463

**Published:** 2024-11-11

**Authors:** Dorien Beeres, Maria Rosaria Galanti, Maria Nilsson, Anni-Maria Pulkki-Brännström

**Affiliations:** 1 Department of Global Public Health, Karolinska Institutet, Stockholm, Sweden; 2 Department of Public Health, Erasmus MC, Rotterdam, Netherlands; 3 Centre for Epidemiology and Community Medicine, Stockholm Region, Stockholm, Sweden; 4 Department of Epidemiology and Global Health, Umeå University, Umeå, Sweden

**Keywords:** INEQUALITIES, SMOKING, ADOLESCENT, PREVENTION

## Abstract

**Background:**

As prevalence of tobacco use falls, socioeconomic inequalities in tobacco use are increasing in many high-income countries. Evidence is lacking on the effect of preventive interventions on socioeconomic inequalities in smoking initiation among adolescents. We evaluated whether a multicomponent school-based prevention programme with parental involvement has differential effects on smoking initiation across socioeconomic groups and affects the magnitude of socioeconomic inequalities in smoking initiation.

**Methods:**

A secondary analysis of data from a 3-year cluster randomised controlled trial, the TOPAS study, conducted in Sweden from 2018 to 2021. Schools were randomised either to the full programme (Tobacco-Free Duo, T-DUO) or minimal intervention (EDU). The analysis was conducted according to intention to treat for the primary outcome, the probability of remaining a non-user of cigarettes at the end of compulsory school (ages 15–16). Parents’ educational attainment was the socioeconomic variable. Differential effects were analysed by comparing adolescents exposed to T-DUO with those exposed to EDU within each socioeconomic group. The effect of the intervention on the magnitude of inequalities was analysed by comparing several measures of absolute and relative inequalities between T-DUO and EDU.

**Results:**

At the end of follow-up, the full programme had a similar, at most moderate effect on smoking initiation in all socioeconomic groups (relative risk 1.13 (95% CI 1.02 to 1.25) in the middle group). The programme did not significantly affect the magnitude of inequalities (Slope Index of Inequality difference 1.49 (95% CI −15.34 to 18.32)).

**Discussion:**

Socioeconomic inequalities in smoking initiation remain substantial. Our results indicate the absence of an effect of the programme T-DUO on these inequalities.

**WHAT IS ALREADY KNOWN ON THIS TOPIC**

- Adolescence is a critical period for forming tobacco use habits. Some previous studies suggest school-based programmes with parental involvement may prevent smoking initiation among adolescents.

**WHAT THIS STUDY ADDS**

- The Tobacco-Free Duo multicomponent school-based intervention with parental involvement neither creates nor reduces, socioeconomic inequalities in smoking initiation among adolescents in Sweden.

**HOW THIS STUDY MIGHT AFFECT RESEARCH, PRACTICE OR POLICY**

- The emergence of socioeconomic inequalities in smoking already in adolescence is a public health concern. Evaluation of preventive programmes should assess equity impacts alongside effects on average outcomes.

## Introduction

 Socioeconomic differences in smoking are responsible for a large proportion of lifestyle-related health inequalities in most high-income countries.^1^ Socioeconomic inequalities in smoking behaviour persist or even increase despite a declining trend in both cigarette uptake and the prevalence of daily cigarette smoking in multiple high-income countries. Studies in Denmark,^2^ Finland,^3^ the UK^4^ and France^1^ found that despite a substantial decline in cigarette use among adolescents over the past 10–20 years, socioeconomically disadvantaged adolescents are consistently more prone to engage in (daily) cigarette smoking. Reducing smoking-related health inequalities and reversing the trend of increasing socioeconomic inequalities in adolescent smoking are public health priorities.^1^,^4^

Risk factors for tobacco initiation and inequalities in tobacco use are attributable to multiple causes. To understand adolescent smoking behaviour, one must consider the multiple environmental contexts adolescents are embedded in, such as family, school and peer relationships, which all have their particular influences, rules and norms. School environment and parental relationships are identified as important mediating factors in the associative pathway between socioeconomic status (SES) and smoking uptake.^5^ A Europe-wide study summarising findings from 35 countries concluded that the association between SES and adolescent weekly smoking was largely explained by an unequal distribution of family-related and school-related factors and to a lesser extent peer-related factors.^6^ Given the important role of psychosocial factors in smoking uptake and socioeconomic disparities, interventions that target these factors might benefit but can also, unintentionally worsen inequalities.

The idea that interventions may fail to address inequalities—and may even exacerbate them—has been discussed within the framework of the ‘inverse prevention law’.^7^ This concept suggests that those who would benefit the most from preventive measures are often the least likely to receive them, causing ‘intervention-generated inequalities’. A number of studies have investigated which types of interventions are most effective in reducing inequalities and which types generate them instead.^8^ In general, downstream, agency-based interventions, that rely on individual participation for behavioural change and are first adopted by individuals with higher SES, are more likely to increase inequalities.^9^ When the targeted behaviour—such as smoking—is rare and primarily concentrated among more vulnerable groups, the selective reach to ‘lower-risk individuals’ presents a significant concern. Interventions that target behaviours contributing to health inequalities should therefore be upstream, structural and affect the entire population equally or even target the more vulnerable groups.^10^ However, interventions that target vulnerable groups, for example, by focusing on deprived areas have not consistently been successful in reducing health inequalities.^5^ A systematic review evaluating the socioeconomic gradients in the effects of universal school-based health behaviour interventions found that interventions based on education only had an overall negative impact on equality measures, while interventions primarily based on environmental change were more likely to narrow inequalities.^5^ Relative to the size of the knowledge gap, surprisingly few intervention studies include a health equity impact assessment.^5 6 8^

Tobacco-Free Duo (T-DUO) is a school-based intervention with parental involvement implemented since the 1990s. The overall aim is to prevent adolescents, aged 12–16 years, from starting to use tobacco. T-DUO aims at creating a protective tobacco-free environment with schools as the main arena for activities that mobilises antitobacco practices and attitudes.^11^ In an experimental study conducted from 2018 to 2021, six components of T-DUO were manualised and the full programme (T-DUO) was evaluated against a minimal intervention consisting of structured classroom education (EDU) (acronym: TOPAS study). An intention-to-treat (ITT) analysis found marginal effects of T-DUO on the prevention of smoking initiation after 2 years of intervention (probability to remain a non-user of cigarettes was 3% higher in the intervention group (relative risk (RR)=1.03 (95% CI 0.98 to 1.08)).^12^ An as-treated analysis of the effect after 3 years (ninth grade, ages 15–16) of the core component—a contract between the adolescent and a significant adult to remain tobacco-free until the end of compulsory school—found a higher proportion of non-smokers among adolescents in a stable contract (RR=1.15 (95% CI 1.04 to 1.26)) compared with adolescents without a contract at all.^13^ However, boys, previous users of any type of tobacco and adolescents with smoking friends and/or smoking parents were substantially less likely to make a contract in the first place.^13^ This effect of the contract among adolescents who are already at lower risk of smoking initiation gives grounds for concern about the possible negative equity impact of the intervention. On the other hand, an opposite hypothesis of positive equity impact is also possible. A universal approach was used in T-DUO with the potential to reach all students before tobacco use was initiated. The programme aimed at strengthening the protective role of the school being a tobacco-free environment. Evidence from previous systematic reviews suggests that environment change components in interventions for adolescents are associated with reduced inequality.^5 8^

The aim of the current study was to evaluate the effect of the programme T-DUO on socioeconomic inequality in smoking initiation. The following research questions were formulated: (1) was the effect of T-DUO on the prevention of smoking initiation (probability to remain a non-user of cigarettes after 3 years of intervention compared with EDU) different between socioeconomic groups (differential effects) and (2) did the magnitude of socioeconomic inequality in smoking initiation differ between T-DUO and EDU at the end of the 3-year intervention period? The study protocol has been previously published.^14^ Research question (1) was retrospectively formulated.

## Methods

### Design

This is a secondary analysis of data from a cluster randomised controlled trial (the TOPAS study) in Sweden.^11^ We applied an ITT approach to compare adolescents in schools randomised to the full programme (T-DUO) with those randomised to a minimal intervention (EDU).

#### Interventions

The interventions were implemented at school by regular school staff. The multicomponent intervention (T-DUO) consisted of six components implemented during the final 3 years of compulsory school when the adolescents were 12–16 years old. The first two components involved informing parents and adolescents about T-DUO at the beginning of seventh grade. The core component was a contract signed by the adolescent and a significant adult whereby both commit to remain tobacco-free for at least 3 years, that is, until the adolescent finishes compulsory school. The three other components were as follows: membership card, which entitled adolescents with the contract to small monetary or in-kind benefits; disclosure of the pair’s tobacco-free status accompanied by a prize draw at the end of each school year and structured classroom education with two interactive lessons per semester until the end of ninth grade. The minimal intervention (EDU) only consisted of this latter component (structured classroom education). The study team provided the intervention materials, including lesson plans, and arranged an annual training and networking day for school staff.

#### Data collection

Adolescent data were collected through paper questionnaires completed at school at baseline (before intervention, ages 12–13, May–August 2018), at 1-year follow-up (May–June 2019), at 2-year follow-up (May–June 2020) and at endline (follow-up 3, ages 15–16, May–June 2021). Here, we used information from baseline and endline only. Information on caregivers’ (later referred to as parents) characteristics was collected through paper-based postal caregivers’ questionnaires administered at the same time points.

#### Health outcome

We analysed the trial’s primary outcome, never tried cigarettes at the end of compulsory school (ninth grade, ages 15–16), which was the end-point of the 3-year intervention period. Cigarette smoking initiation was self-reported by answering the question ‘Have you ever tried smoking a cigarette, even if it was just a few puffs?’ with response alternatives: ‘no, never tried’; ‘yes’. Adolescents who answered ‘no, never tried’ were classified as ‘never cigarette smokers’. We dropped a planned analysis of adult smoking cessation, which was a trial secondary outcome, due to a low response rate among parents to the smoking question in the follow-up, which constitutes a deviation from the published protocol.^11 14^

#### Socioeconomic variable

The highest educational level attained by the adolescents’ parents was used to categorise adolescents by socioeconomic group. Educational attainment was self-reported by parents in the baseline caregivers’ questionnaire. If baseline information was missing, we used information from one of the annual follow-up questionnaires. Education was categorised using the International Standard Classification of Educational (ISCE) levels. We used a primary categorisation in three groups, taking into account that most adolescents had two parents: ‘no parents with 12 or more years of education’ (no university degree, ISCE 1–5; lowest socioeconomic group); ‘one parent with 12 or more years of education’ (at least bachelor’s or equivalent level, ISCE 6 or more; middle socioeconomic group) and ‘both parents with 12 or more years of education’ (highest socioeconomic group). For the sake of comparability with previously published analyses of the TOPAS study, we also used a binary categorisation as ‘no parents with 12 or more years of education’ and ‘at least one parent with 12 or more years of education’.

#### Covariates

Because we kept to the initial randomisation and did not observe large differences in the distribution of baseline characteristics between socioeconomic groups, in line with previously published ITT results, no covariate adjustment was done. However, we present the following covariates available in the questionnaires to describe the sample, selected based on the framework of the WHO Commission on the Social Determinants of Health.^15^ Adolescent questionnaire: Adolescent’s gender was self-reported as male/female/other or I don’t want to say. The following two questions were used to define smoking friends: (1) Of the friends you spend most of your free time with, how many smoke cigarettes? ‘None, less than half, about half, more than half, all, I don’t know’. (2) Do some of your closest friends smoke? ‘Smoke daily, smoke sometimes, don’t smoke, don’t know, don’t see them/don’t have them’. Adolescents who answered affirmatively to the first question (less than half—all) or second question (smoke daily, smoke sometimes) were classified as having smoking friends.

Parental questionnaire: Parents’ migrant background was measured as born or not in Sweden. We also included information on whether the adolescent was living with both parents and whether both parents were in employment. As other intermediary determinants, we included parents’ self-reported smoking status, and those who reported that they smoke regularly or only occasionally were categorised as current smokers.

### Statistical analysis

In order to study the probability to remain a non-user of cigarettes, we excluded adolescents who had already tried smoking at baseline (ages 12–13) from the analytical sample. We conducted a complete case analysis and thus excluded adolescents with missing data on smoking status at baseline or at endline (online supplemental appendix A).

To answer the first research question, we first compared the prevalence of never smoking between socioeconomic groups by computing prevalence differences with 95% CIs. We then estimated the intervention effect within each SES using generalised linear models, Poisson family and log link and robust SEs. As a complement to the stratified analysis, we did an interaction analysis including socioeconomic group and intervention group.

To answer the second research question, we first computed the Slope Index of Inequality (SII) (with 95% CIs). We considered absolute inequality as measured by SII to be of primary policy importance as also argued by others.^16^ SII is a summary measure of the linear association between the socioeconomic variable and the health outcome that takes into account the relative size of each socioeconomic group by using a rank variable, which enables comparisons across populations and outcomes.^17^ The rank variable *x(k*) is defined as the population in strictly higher educational groups+half of the population in group *k*. Second, we computed the Relative Index of Inequality (RII), a summary measure analogous to the SII that captures relative rather than absolute inequality. A log-linear model was used to estimate the form *f*_β_(x)=y_0_exp(βx) and where RII=exp(β*).^18^ Generalised linear models, binomial regressions with, respectively, identity and log link were used to estimate the SII and RII as advised for outcomes that are common.^18^ If the model did not converge, Poisson regression with a log link was used. Sandwich estimators were used to compute the 95% CI. Finally, we compared each measure between T-DUO and EDU to test the null hypothesis that socioeconomic inequality in smoking initiation was no different between these two groups. The command syntax can be found at **https://osf.io/hf8sr/**.

## Results

### Participants and response rate

A total of 34 schools were included in the trial, with 17 randomised to T-DUO and 17 to EDU, involving 1924 eligible adolescents. Of these, 1776 adolescents answered the questionnaire at baseline, of whom 1413 (79.6%) answered the same questionnaire at endline. We excluded 30 adolescents who had already tried cigarettes at baseline, and 4 who had missing information on baseline cigarette smoking. Information about parents’ education was missing for 180 (13%) and information on their own smoking at endline was missing for 19 adolescents (1.3%). Thus, the final analytical sample consisted of 1180 adolescents with complete data (online supplemental appendix A). Adolescents with two higher-educated parents (highest socioeconomic group) had a higher response rate at endline (retainment=86.9%) compared with those with one high-educated parent (retainment=81.2%) and those with lower educated parents (retainment=78.2%).

#### Comparison of baseline characteristics between T-DUO and EDU

The distribution of baseline characteristics by socioeconomic group is presented in table 1. Overall, three-quarters of adolescents had one or more higher-educated parents. The proportions of adolescents in each of the three socioeconomic groups were very similar for T-DUO and EDU. Adolescents in the lowest socioeconomic group were less likely to have both parents in employment, more likely to have parents who currently use tobacco (cigarettes or snus) and more likely to have smoking friends, compared with adolescents in the highest socioeconomic group. These socioeconomic patterns in the potential predictors of smoking were very similar between T-DUO and EDU.

**Table 1:** Baseline characteristics of the analytical sample (cigarette naïve adolescents)

		T-DUO (N=573)			EDU (N=607)	
Parents’ educational attainment	None 12 years	One ≥12 years	Both≥12 years	None 12 years	One ≥12 years	Both ≥12 years
	136 (23.7%)	212 (36.9%)	225 (39.3%)	145 (23.9%)	218 (35.9%)	244 (40.2%)
Adolescent’s gender						
Female	69 (50.7%)	103 (48.6%)	112 (49.8%)	77 (53.1%)	120 (55.0%)	117 (48.0%)
Male	66 (48.5%)	105 (49.5%)	109 (48.4%)	67 (46.2%)	95 (43.6%)	122 (50.0%)
Other/prefer not to say	1 (0.7%)	4 (1.9%)	2 (0.9%)	1 (0.7%)	3 (1.4%)	5 (2.0%)
Both parents employed (yes %)	123 (90.4%)	201 (94.8%)	214 (95.1%)	132 (91.0%)	189 (86.7%)	229 (93.9%)
Living with both parents (yes %)	102 (75.0%)	161 (75.9%)	190 (84.4%)	110 (75.9%)	162 (74.3%)	204 (83.6%)
Parent born abroad (yes %)	21 (15.4%)	43 (20.3%)	37 (16.4%)	30 (20.7%)	45 (20.6%)	47 (19.3%)
Current smoking parent (yes %)	13 (9.6%)	15 (7.1%)	6 (2.7%)	19 (13.1%)	14 (6.4%)	10 (4.1%)
Current snus use parent (yes %)	14 (10.3%)	15 (7.1%)	14 (6.2%)	22 (15.2%)	14 (6.4%)	23 (9.4%)
Smoking friends (yes %)	19 (14.0%)	14 (6.6%)	17 (7.6%)	27 (18.6%)	26 (11.9%)	19 (7.8%)

T-DUOTobacco-Free Duo

#### Intervention effect on the probability to remain a non-user of cigarettes

At the end of follow-up, the probability of remaining cigarette naïve was lowest among adolescents in the lowest socioeconomic group (73.5% in T-DUO, 70.3% in EDU). Comparing T-DUO and EDU, the probability was higher in T-DUO in all socioeconomic groups with the largest difference in the middle socioeconomic group (RD 9.64 (95% CI 1.96 to 17.32) points higher prevalence in T-DUO) (table 2). The association of the intervention with the probability to remain a non-smoker varied across socioeconomic groups and we indicated a possible intervention effect in the two highest socioeconomic groups, but not in the lowest (lowest SES group RR 1.05 (95% CI 0.90 to 1.21); middle SES group RR 1.13 (95% CI 1.02 to 1.25); highest SES group RR 1.08 (95% CI 1.00 to 1.17)). Using the binary categorisation of parental education yielded similar results, but the interaction between socioeconomic group and intervention effect was not statistically significant (table 2).

**Table 2:** Intervention effects by socioeconomic group

	Prevalence of cigarette naïve adolescents at end of follow-up				
	**T-DUO**	**EDU**	**T-DUO vs EDU**	**T-DUO vs EDU**	
Parents’ educational attainment (three categories)			Risk difference(95% CI)	Intervention effect (95% CI)	Interaction Intervention and parental education
None 12 years	73.5%	70.3%	3.18 (−7.32, 13.68)	1.05 (0.90, 1.21)	
One ≥12 years	83.5%	73.9%	9.64 (1.96, 17.32)	1.13 (1.02, 1.25)	1.08 (0.91, 1.29)
Both ≥12 years	88.0%	81.6%	6.44 (−0.02, 12.90)	1.08 (1.00,1.17)	1.03 (0.88, 1.22)
Parents’ educational attainment (2 categories)					
None 12 years	73.5%	70.3%	3.2 (−7.3,13.7)	1.05 (0.90, 1.21)	
At least one ≥12 years	85.8%	77.9%	7.0 (2.9,12.9)	1.10 (1.04, 1.17)	1.05 (0.90,1.23)

T-DUOTobacco-Free Duo

### Socioeconomic inequalities in smoking initiation

At the end of the follow-up, we found socioeconomic inequalities in adolescent smoking initiation but no difference in the size of inequalities between T-DUO and EDU (table 3). The prevalence difference between the lowest and highest socioeconomic groups was −14.47 (95% CI −23.02 to –5.93) in T-DUO and −11.21 (95% CI −20.10 to −2.33) in EDU, and SII was −18.78 (95% CI −30.05 to –7.51) in T-DUO and −17.29 (95% CI −29.79 to −4.78) in EDU. Comparing T-DUO and EDU, these measures of absolute inequality were similar in size (prevalence difference: 3.26 (95% CI −9.07 to 15.59), SII difference 1.49 (95% CI −15.34 to 18.32)). Regarding relative inequality, the prevalence ratio and RII were remarkably similar in size between T-DUO and EDU. Using the binary categorisation of SES yielded similar results (table 3).

**Table 3:** Socioeconomic inequalities by intervention group

	Socioeconomic inequalities		
	**T-DUO**	**EDU**	**T-DUO vs EDU**
Parents’ educational attainment (three categories)			
Prevalence difference (none vs both ≥12 years)	−14.47 (−23.02,−5.93)	−11.21 (−20.10,−2.33)	3.26 (−9.07,15.59)
Prevalence ratio (none vs both ≥12 years)	0.84 (0.75,0.93)	0.86 (0.76,0.97)	1.03 (0.88,1.22)
Slope Index of Inequality (SII)	−18.78 (−30.05,−7.51)	−17.29 (−29.79,−4.78)	1.49 (−15.34,18.32)
Relative Index of Inequality (RII)	0.79 (0.68,0.91)	0.80 (0.68,0.94)	1.01 (0.81,1.27)
Parents’ educational attainment (two categories)			
Prevalence difference	−12.28(−20.39,−4.18)	−7.58 (−15.92,0.76)	4.71 (−6.92,16.34)
Prevalence ratio	0.86 (0.77,0.95)	0.90 (0.80,1.01)	1.05 (0.90,1.23)
SII	−24.6 (−40.8,−8.3)	−15.2 (−30.2,−1.5)	9.4 (−13.8,32.7)
RII	0.73 (0.59,0.91)	0.81 (0.65,1.03)	1.11 (0.81,1.52)

T-DUOTobacco-Free Duo

Parental smoking, parental snus use and smoking friends were unbalanced at baseline. We, therefore, repeated the analysis described above adding the unbalanced covariates, as a sensitivity analysis. Adding parental snus use and smoking friends improved model fit (Akaike information criterion and Bayesian information criterion). Adding parental smoking did not improve model fit. The adjusted models indicated less socioeconomic inequality in EDU but overall the strength and direction of the estimates from the adjusted models were comparable to the estimates presented as main findings (online supplemental appendix B). The models’ fit and the results did not change when the unbalanced variables were entered separately.

## Discussion

This secondary analysis of a cluster randomised trial indicated no effect of a multicomponent school-based intervention with parental involvement (T-DUO) on emerging socioeconomic inequalities in smoking initiation among adolescents during a 3-year period, while this behaviour still recognises substantial socioeconomic differences. By evaluating the equity impact of an intervention, this study contributes to the surprisingly sparse empirical evidence of what effect public health interventions have on socioeconomic inequalities in health. In public health, SES has for many years been of key interest with empirical studies tending to assess SES as a determinant of health status. In evaluation studies, analysis of average effects is increasingly (though not always) complemented by analysis of whether intervention effects vary between groups, for example, women/men or SES. Such analyses of differential effects are very important for understanding for which groups a given intervention works or does not work. In the present paper, we additionally analyse the magnitude of inequality as the outcome of interest. In such analyses a positive intervention effect reduces the magnitude of inequality, a negative effect increases it and no intervention effect indicates that the presence and magnitude of inequality remain unchanged. In this study we found no intervention effect on inequality, despite indications of stronger intervention effects among children of better-educated parents compared with those of less-educated parents.

The neutral equity impact observed in this study adds to the previous evidence that suggests universal school-based prevention interventions often have no effect on inequality, or findings are mixed or unclear.^5 19^ However, it contrasts with the recent evaluation of the X:IT II intervention, an intervention similar to T-DUO, which reported diminished socioeconomic disparities in smoking over 2 years.^20^ A recent review of subgroup effects of interventions targeting adolescent multiple risk behaviour found that, based on four studies, the direction of effect favoured lower socioeconomic groups but not convincingly so (RR 0.83 (95% CI 0.66 to 1.03).^21^ Tobacco taxation has been identified as one of the few successful policies in reducing tobacco-related inequalities among the population at large^10^ as well as specifically among adolescents.^19^ More studies on the equity impact of tobacco prevention programmes are needed to be able to put our findings in a broader perspective.

T-DUO is a universal approach to tobacco prevention that targets all adolescents on equal terms. Due to individual-level and school-level factors, all adolescents may not benefit from the intervention in practice. First, adolescents with smoking parents may find it more difficult to identify a significant adult with whom to sign the contract. To counteract such difficulties, the choice of an adult partner is up to the adolescent themselves. Second, schools’ own reporting revealed considerable differences in the degree of implementation and only a minority of schools implemented all six programme components.^22^ Such individual-level and school-level factors may create unequal access to the intervention not directly considered in the current analysis.

The present study has two other limitations. A lower sample size in the lowest socioeconomic group and loss to follow-up may have resulted in a selected sample of adolescents from low-risk families, which could have resulted in an underestimation of the magnitude of socioeconomic inequalities in smoking initiation. Finally, the TOPAS study was not designed to detect health equity impacts and a lack of statistical power is another possible explanation for why we did not detect differences in effect size between socioeconomic groups. The sample size calculation was based on an average effect size of 1.10 (risk ratio intervention to control) between T-DUO and EDU^11^ which is similar in magnitude to the highest effect size observed here (RR 1.13, 95% CI 1.02 to 1.25, middle socioeconomic group). Difficulties to obtain written informed consent from guardians affected the recruitment rate, and the 1924 adolescents at baseline were well below the intended 3000 based on the sample size calculation.^22^

To conclude, we found no effect of T-DUO on socioeconomic differences in smoking initiation among adolescents. Our findings concern a selected sample and future research should strive to include more diverse samples. We hope our analysis encourages other evaluators to conduct similar studies in the future to allow for meaningful meta-analyses of the equity impact of public health interventions.

## supplementary material

10.1136/jech-2024-222463 online supplemental file 1

## Data Availability

Data are available on reasonable request.
