# Supplementary material for: Effect of a multicomponent school-based intervention with parental involvement on socioeconomic inequalities in smoking initiation: equity impact analysis of the TOPAS study
Source: J Epidemiol Community Health. 2024 Nov 11;79(3):e222463. doi: 10.1136/jech-2024-222463 (PMC11874375; doi:10.1136/jech-2024-222463)
Supplement: online supplemental file 1 [file jech-79-3-s001.pdf]

Begränsad delning

Appendix A. Analytical sample.

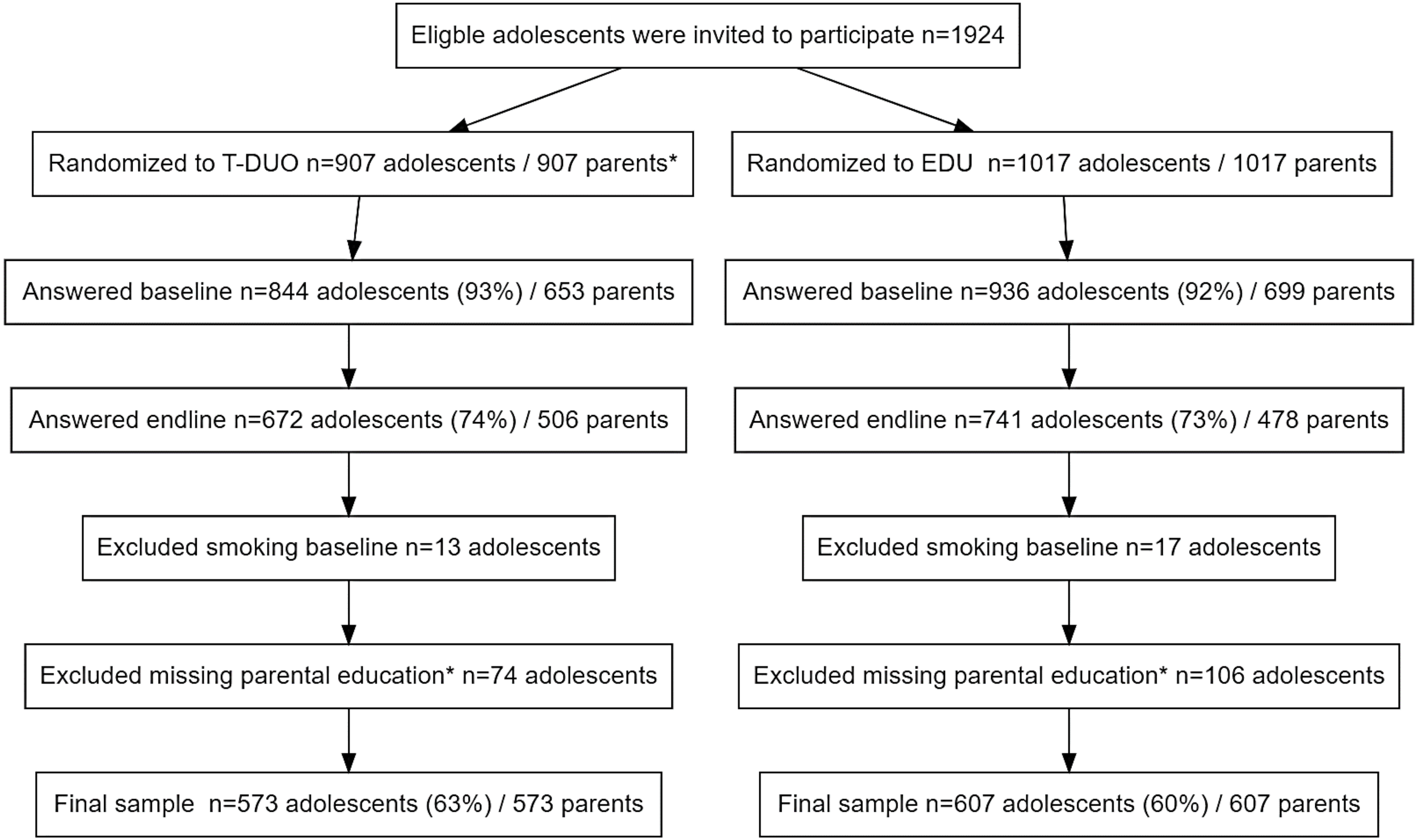

Notes to Figure. * Parents include caregivers.

Appendix B. Sensitivity analysis of socioeconomic inequalities by intervention group

	Socioeconomic inequalities[1]		
	**T-DUO**	**EDU**	**T-DUO vs EDU**
**Parents' educational attainment (3 categories)**			
Prevalence Difference *(none vs both ≥12 years)*	-11.52 [-20.87,-2.17]	-5.48 [-14.68,3.72]	7.48 [-5.63,20.59]
Prevalence ratio *(none vs both ≥12 years)*	0.85 [0.75,0.97]	0.92 [0.82,1.04]	1.10 [0.93,1.31]
Slope index of inequality (SII)	-13.80 [-26.00,-1.60]	-11.80 [-24.21, 1.85]	4.19 [-13.66,22.05]
Relative index of inequality (RII)	0.82 [0.70,0.95]	0.86 [0.72,1.02]	1.08 [0.85,1.36]
**Parents' educational attainment (2 categories)**			
Prevalence difference	-10.25 [-18.34,-2.17]	-1.74 [-10.43,6.94]	10.27 [-1.20,22.73]
Prevalence ratio	0.87 [0.77,0.98]	0.94 [0.84,1.06]	1.10 [0.80,1.51]
Slope index of inequality (SII)	-20.51 [-36.68,-4.33]	-9.13 [-25.89,7.62]	14.64 [-4.26,18.9]
Relative index of inequality (RII)	0.76 [0.61,0.95]	0.94 [0.84,1.06]	1.21 [0.58,2.08]

[1] All models are controlled for parental snus use and smoking friends.
